# A voyage to Terra Australis: human-mediated dispersal of cats

**DOI:** 10.1186/s12862-015-0542-7

**Published:** 2015-12-04

**Authors:** K. Koch, D. Algar, J. B. Searle, M. Pfenninger, K. Schwenk

**Affiliations:** Biodiversity and Climate Research Centre (BiK-F) by Senckenberg Naturforschende Gesellschaft and Goethe-University, Senckenberganlage 25, 60325 Frankfurt am Main, Germany; Department of Parks and Wildlife, Science and Conservation Division, P.O. Box 51, Wanneroo, 6065 WA Australia; Department of Ecology and Evolutionary Biology, Cornell University, Corson Hall, Cornell University, Ithaca, NY 14853-2701 USA; Molecular Ecology, Institute for Environmental Sciences, Universität Koblenz-Landau, 76829 Landau in der Pfalz, Germany

**Keywords:** *Felis silvestris catus*, Human introduction, Commensal, Feral cat, Phylogeography, Colonization history

## Abstract

**Background:**

Cats have been transported as human commensals worldwide giving rise to many feral populations. In Australia, feral cats have caused decline and extinction of native mammals, but their time of introduction and origin is unclear. Here, we investigate hypotheses of cat arrival pre- or post-European settlement, and the potential for admixture between cats of different invasion events. We analyse the genetic structure and diversity of feral cats from six locations on mainland Australia, seven Australian islands and samples from Southeast Asia and Europe using microsatellite and mitochondrial DNA data.

**Results:**

Our results based on phylogeographic model selection are consistent with a European origin of cats in Australia. We find genetic distinctiveness of Australian mainland samples compared with Dirk Hartog Island, Flinders Island, Tasman Island and Cocos (Keeling) Island samples, and genetic similarities between some of the island populations. Historical records suggest that introduction of cats to these islands occurred at the time of European exploration and/or in connection with the pearling, whaling and sealing trades early in the 19th century. On-going influx of domestic cats into the feral cat population is apparently causing the Australian mainland populations to be genetically differentiated from those island populations, which likely are remnants of the historically introduced cat genotypes.

**Conclusion:**

A mainly European origin of feral cats in Australia, with possible secondary introductions from Asia following the initial establishment of cats in Australia is reasonable. The islands surrounding Australia may represent founding populations and are of particular interest. The results of the study provide an important timeframe for the impact of feral cats on native species in Australia.

**Electronic supplementary material:**

The online version of this article (doi:10.1186/s12862-015-0542-7) contains supplementary material, which is available to authorized users.

## Background

The deliberate or accidental translocation of species from their native habitat to new environments by humans [1, 2] may trigger substantial environmental effects [3, 4]. The consequences for native biodiversity and their economic impact have been a focus of many ecological and conservation-related studies [1, 5]. For example, approximately 40 % of the species listed as threatened or endangered under the Endangered Species Act of the US are considered to be primarily at risk through competition with or predation by invasive species [6]. The economic costs caused by invasive species through environmental damage and losses are perceived as a major concern throughout the world [7]. Considering Australia, the focus of this study, the economic impact of 11 key vertebrate pest species has been calculated at 720 million Australian dollars per annum [8]. The introduction of invasive species, historical changes in land use and habitat transformation in Australia have led to the extinction of approximately 22 terrestrial vertebrate species and a critically endangered status for 43 others [9, 10].

Over the last few hundred years the number of species invasions has increased by orders of magnitude worldwide [4, 11]. This is true of Australia [8] for which two of the 22 invasive mammalian species are predators: the European red fox (*Vulpes vulpes*) and the domestic cat (*Felis silvestris catus*) [12]. Cats are the subject of this paper and as invasive domesticates may be grouped into three categories: (1) *Feral cats* are defined as free-living, independent of humans and reproducing in self-perpetuating populations. (2) *Stray cats,* rely to some extent on human provision (typically rubbish tips). These were originally housecats that went astray and now refrain from human contact. (3) *Housecats* and fancy breed cats that depend almost entirely on humans [13].

Feral cats have established long-lasting and stable invasive populations over large geographical areas. There is strong evidence that cats have a detrimental impact on native wildlife, driving the declines of bird, mammal and reptile species [14, 15]. Attempts to reintroduce threatened species in the arid zone of Australia have often failed because of extensive predation by feral cats [16, 17].

Although feral cats do not depend on humans, they derive from cats that did; and the genetic equivalency is informative. Therefore, the current populations of cats may, through genetic analysis [18], allow inferences about the mostly maritime introduction history and routes. Cats were likely domesticated around 11,000 years ago in the Near East, perhaps first associating with early human settlements because the presence of rodent pests provided prey opportunities, and becoming a companion animal thereafter [19, 20]. Once domesticated, cats would have been moved around by humans. Especially since 1800, domestic cats have actively been transported on sailing vessels of explorers, sealers, whalers and colonists either for controlling rodents or as pets - first on board and later in new settlements [13, 21] leading to the global distribution of cats covering most continents and even remote islands [19, 21, 22]. Feral cats can thus derive from this deliberate transport, or from cats that were transported accidentally - which may have happened quite frequently with large ships.

The origin and sequence of invasions of cats into Australia is unresolved [13, 23–25]. It would be reasonable to suggest that cats may be a relatively recent introduction with European explorers and settlers in the late 18th century [13, 23]. Another alternative assumes cat arrival to Australia prior to European settlement from (i) shipwrecks in Western Australia around 1600, (ii) or with Malaysian trepangers from about 1650 in northern Australia, (iii) or even earlier with the introduction of the dingo (*Canis lupus dingo*) around 4500 years ago (which, of course, goes against the normal assumption of arrival of cats in Australia within the last few hundred years) [23, 25–28].

Admixture of putative cat source populations is almost certain to have been of importance. Even if cats were first introduced from Asia, it is likely that there has been interbreeding with cats from secondary multiple introductions at various occasions by European settlers [23] and continuously since then through stray housecats from mixed geographic origin (Australian Social Trends, 1995, Australian Bureau of Statistics). In order to unravel the evolutionary history and dispersal patterns of Australian feral cats, we applied a phylogeographic approach covering the Australian mainland and offshore islands. Theoretical and empirical studies have shown that hybridization and intermixing through multiple introductions is less likely to occur on islands than in comparable mainland populations [29]. Thus, island populations may be genetically representative of the original founder population.

We therefore analysed samples from six mainland and seven island locations including Australian Indian Ocean Territories (Christmas Island and Cocos (Keeling) Island; hereafter referred to as Cocos Island) as well as samples from Southeast Asia and previously published data from Europe [19]. Christmas and Cocos Islands were inhabited by European merchants during the time of exploration and settlement of Australia [30, 31]. Microsatellite and mitochondrial DNA data were used to address the following specific questions: (i) Do Australian cat populations share alleles and haplotypes with European and/or Asian populations? (ii) What is the regional genetic population structure and how many genetically distinct groups exist across Australia? We discuss our findings in the context of historical reports about the early exploration and settlements in the Australasian region. We follow a phylogeographic approach for the study of human-mediated movement of commensals, domesticates and other species closely associated with people [27].

## Results

### Genetic population structure and differentiation using microsatellites

We genotyped 269 individuals representing 14 sampling locations at 12 microsatellite loci, one of which was excluded because of null alleles (F85) [32]. For the remaining 11 loci the expected heterozygosity was high, ranging from 0.63 to 0.88 (mean *H*_*E*_ = 0.76). Australian mainland locations exhibited the highest genetic diversity (Table 1). The mean number of alleles per locus varied greatly between populations ranging from 4.8 to 12.1 (Table 1). Christmas Island (CIF) and the Australian mainland location in the south, Cape Arid (CA), had the largest number of alleles with 11.8 and 12.1, respectively. The observed heterozygosity was always lower than the expected heterozygosity (Table 1) reflecting presumably a degree of inbreeding or population substructure.

**Table 1:** Microsatellite statistics of Australian and Southeast Asian populations

Group	Population (abbreviation)	*N*	*NA*	*H*_*O*_	*H*_*E*_	*F*_*IS*_	*PA*	*PA/N*
Territorial Islands	Christmas Island (CIF)	79	11.8	0.63	0.74	0.11	18	0.22
	Cocos (Keeling) Island (Q)	42	7.0	0.51	0.63	0.18	6	0.14
W Australia - mainland	Kimberley (KIM)^a^	5	-	-	-	-	-	-
	Cape Arid National Park (CA)	23	12.1	0.79	0.88	0.10	14	0.60
	Mount Keith (MK)^a^	8	-	-	-	-	-	-
	Fitzgerald National Park (FG)	10	7.4	0.77	0.82	0.06	3	0.30
	Peron (PE)	13	6.9	0.75	0.78	0.03	0	0
	Tips South West (TSW)	25	9.8	0.74	0.82	0.10	6	0.24
W Australia - island	Dirk Hartog Island (DHI)	40	9.6	0.72	0.75	0.03	11	0.27
SE Australia - islands	Flinders Island (FL)^a^	3	-	-	-	-	-	-
	French Island (FI)^a^	3	-	-	-	-	-	-
	Tasmania (TAS)	10	4.8	0.70	0.72	0.04	3	0.30
	Tasman Island (TASM)^a^	5	-	-	-	-	-	-
Asia	Malaysia (M)^a^	3	-	-	-	-	-	-
	Total	269						

Statistics for microsatellite typing of cat populations in the Australian mainland, Australian islands and Southeast Asia (based on 11 loci), including population sample size (*N*), mean number of alleles per locus (*NA*), observed (*H*_*O*_) and expected (*H*_*E*_) heterozygosity, inbreeding coefficient (*F*_*IS*_) and number of private alleles per population over all loci (*PA*)

^a^Sample size lower than 10 are not considered for population genetic parameters

**Fig. 1:**
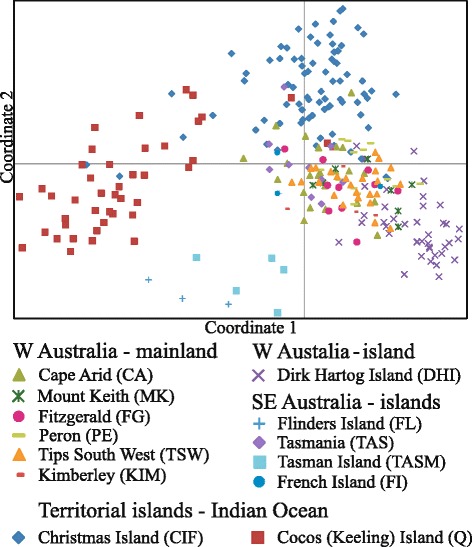
Principal Coordinates Analysis (PCoA). Principal Coordinates Analysis (PCoA) plot using microsatellite data from 13 populations of cats from Australia and surrounding islands

The analysis of pairwise genetic differentiation between populations indicated that Cocos Island and Christmas Island (Q, CIF), the south-eastern islands Tasman Island (TASM) and Flinders Island (FL) as well as the most western island of Australia - Dirk Hartog Island (DHI) - were distinct from all other populations, particularly those on the Australian mainland and Tasmania (PCoA: Fig. 1; 28.5 % and 24.5 % of variation explained by axis 1 and 2, respectively).

**Fig. 2:**
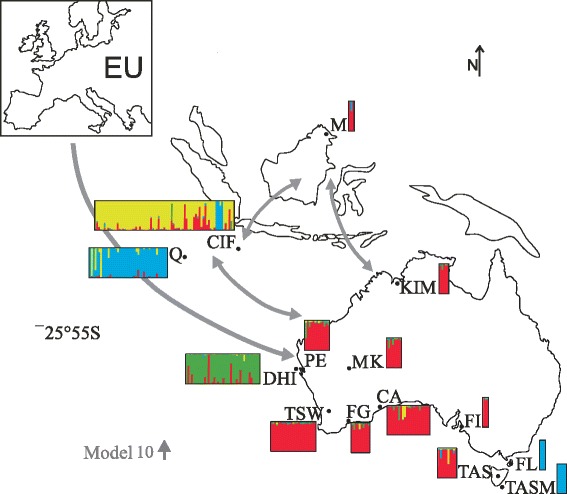
Map of Australia, Southeast Asia and Europe with possible invasion routes. Possible invasion routes of cats shown on a map of Australia and Southeast Asia with Europe (EU) in the top left-hand corner. Arrows indicate invasion routes with highest support from the phylogeographic model selection approach (model 10 grey arrows; further details in Additional file 4: Figure S3). STRUCTURE plots showing ancestry (*K* = 4) inferred from microsatellite data for mainland Australia, Australian islands and Southeast Asia. Each individual cat is represented by a single vertical line in plots for each location. Abbreviations for populations follow Table 1

The Bayesian assignment approach gave *K* = 4 (Additional file 1: Table S1). Individual cluster assignments are shown in Fig. 2 (for reference, results for *K* = 2 and 3 are shown in Additional file 2: Figure S1). This analysis grouped the Australian mainland and Tasmanian samples together with the Southeast Asian samples. Cats from the Tasman, Flinders and Cocos Islands (TASM, FL, Q) had similar ancestry structures, while cats from Christmas Island (CIF) and Dirk Hartog Island (DHI) were distinctive from all other populations (Fig. 2).

The major portion of nuclear genetic variation was found within populations (AMOVA: 67.8 %; *F*_*ST*_ = 0.32; *P* < 0.001) with 7.5 % (*F*_*CT*_ = 0.07; *P* = 0.224) among the four geographic regions (EU, AS, OZ, CIQ) and 24.7 % (*F*_*SC*_ = 0.26; *P* < 0.001) among populations within regions.

### Mitochondrial phylogeography

In total 2603 base pairs of the mitochondrial segment were sequenced. Altogether we detected 63 haplotypes in the dataset of which 25 were present in the European populations (*N* = 39, haplotype diversity = 0.94, nucleotide diversity = 0.0080). All other populations (*N* = 1 – 39) exhibited between 1 to 6 haplotypes, and had haplotype diversities of 0 – 0.80 and nucleotide diversities of 0 – 0.0020.

**Fig. 3:**
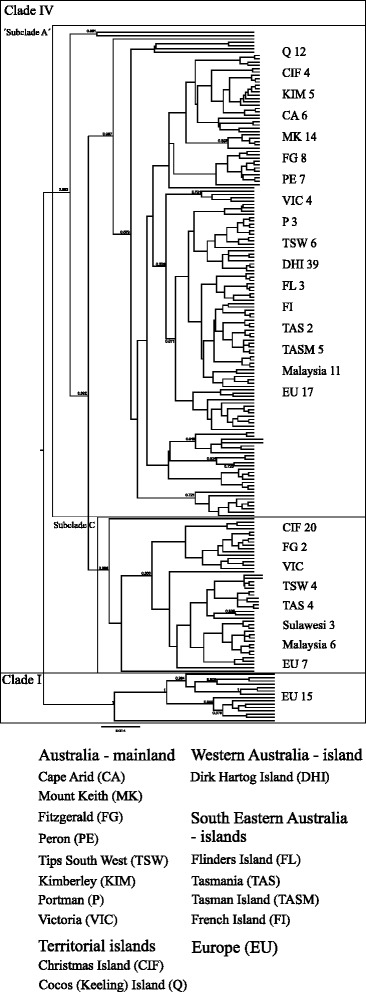
Phylogenetic tree of mtDNA haplotypes. Bayesian phylogenetic tree of mtDNA haplotypes of cats from Australia and Southeast Asia in the context of previously published data (further details in Additional file 3: Figure S2). The nomenclature of clades and subclades follows Driscoll et al. [19]. The numbers of individuals per location are given and the highest posterior density (HPD) represented at the node

The Bayesian phylogenetic tree (Fig. 3) revealed the two major clades described by Driscoll et al. [19] for European cats, clade I and IV (further details in Additional file 3: Figure S2). Clade I only represents the European wildcat sequences from Driscoll et al. All the Australasian samples were of clade IV together with European sequences from Driscoll et al. Within clade IV, subclades A and C of Driscoll et al. can be seen. A small subclade of Australian samples (DHI, TSW, CA) could not be matched with certainty with Driscoll et al.’s subclades. The largest subclade (A) consists of a mixed group of Australian islands (DHI, TASM, FL and TAS) and mainland (PE, MK, FG, CA, KIM, TSW, P, VIC) populations and samples from Southeast Asia, Christmas and Cocos Island and throughout Europe. Subclade C mainly consists of samples from Asia, Christmas Island, Tasmania, Tips South West and western and central Europe.

Pairwise population comparisons showed low genetic differentiation between samples from Europe and elsewhere (mean *F*_*ST*_ of 0.18) and between samples from Asia and elsewhere (mean pairwise *F*_*ST*_ of 0.16; Table 2). Significant strong genetic differentiation to most other populations was found for CIF (except with TAS), for FL (except with TASM) and TASM. Very low genetic differentiation was found between TAS, TSW, CIF and EU with pairwise *F*_*ST*_ values ranging from 0.03 to 0.25.

**Table 2:** Genetic differentiation among populations

	CA	DHI	FG	FL	KIM	MK	PE	TAS	TASM	TSW	CIF	Q	EU	AS
Cape Arid (CA)		*	-	*	-	*	*	-	*	*	*	-	*	-
Dirk Hartog Island (DHI)	0.37		*	-	*	-	-	*	*	*	*	*	*	*
Fitzgerald National Park (FG)	0.12	0.09		*	-	-	-	-	*	-	*	*	*	-
Flinders Island (FL)	0.56	0.34	0.36		*	*	*	-	-	*	*	*	*	*
Kimberley (KIM)	0	0.30	0.05	0.53		*	*	-	*	-	*	-	*	-
Mount Keith (MK)	0.47	0.06	0.07	0.71	0.39		-	*	*	*	*	*	*	*
Peron (PE)	0.50	0.02	0.07	0.78	0.43	0		*	*	*	*	*	*	*
Tasmania (TAS)	0.39	0.56	0.25	0.51	0.36	0.65	0.63		*	-	-	*	-	-
Tasman Island (TASM)	0.64	0.38	0.43	0	0.62	0.74	0.82	0.60		*	*	*	*	*
Tips South West (TSW)	0.24	0.26	0	0.24	0.18	0.30	0.26	0.03	0.39		*	*	*	-
Christmas Island (CIF)	0.66	0.65	0.49	0.67	0.65	0.72	0.71	0.07	0.69	0.25		*	*	*
Cocos (Keeling) Island (Q)	0.17	0.63	0.51	0.88	0.27	0.79	0.84	0.63	0.89	0.53	0.76		*	*
Europe (EU)	0.17	0.28	0.13	0.16	0.15	0.25	0.22	0.09	0.22	0.09	0.23	0.28		*
Malaysia/Sulawesi (AS)	0.07	0.18	0	0.27	0.03	0.19	0.18	0.13	0.32	0	0.37	0.31	0.16	

Genetic differentiation among cat populations based on mtDNA data. In lower matrix pairwise *F*_*ST*_ values are given. In upper matrix asterisks (*) and dashes (-) indicate significant (*P* < 0.05) and non-significant differences, respectively

Comparison of potential invasion routes using the model selection approach supported an invasion of cats from Europe with bidirectional movement between Australia, Christmas and Cocos Island and Southeast Asia (Fig. 2; model 10 in Additional file 1: Table S2, Additional file 4: Figure S3: LnL of -851.35 and AIC of 1724.70).

## Discussion

Overall, our analyses are in line with the hypothesis of an introduction and establishment of cats in Australia from Britain and other Western and Central European locations as documented by Abbott [23, 24]. Abbott inferred multiple coastal introductions between 1804 - 1886, rather than a spread from the earliest point of colonization (Sydney, 1788) [23, 24]. There was no evidence of a separate and stable feral cat population originating solely from Asian locations (e.g. cats that might have been brought in by Malaysian trepangers [23, 26]). We assume a secondary introduction of Asian cats following European colonization indicated by a grouping of Asian locations with Australian samples (phylogenetic tree, Fig. 3) and a low genetic differentiation to Western Australian locations (CA, FG, KIM and TSW; Table 2). There is an indication of bidirectional movement of cats between Australia and Asia additional to the European colonization in the highest supported model (Model 10; Additional file 4: Figure S3). However, caution is needed in inferring the involvement of Asian cats in the history of cat colonization in Australia due to the small number of Asian samples. The second most likely introduction scenario (Model 5; Additional file 4: Figure S3) includes direct introduction of European cats to Asia, while Model 10 does not show this direct introduction. Thus, what we call ‘Asian cats’ here plausibly derive from Europe as well.

The likelihood of survival of a few introduced founder individuals in a foreign environment may be low due to the presence of native or previously introduced predators (e.g. dingos, various snake species in an Australian context) [18] and increased genetic drift and inbreeding [29, 33]. These genetic effects have been documented in a survey of cats of the Kerguelen archipelago [29, 33–35]. In contrast, moderate or even increased levels of genetic variation of founder populations indicate invasion from multiple sources, predisposing successful introduction and long lasting establishment of invasive species [29, 33, 36]. Multiple introductions leading to inter-mixing among individuals from genetically divergent populations may result in higher genetic variation in founder populations than in original populations [29]. Overall genetic diversity levels in Australian cats was found to be similar to that of European domestic cats (*H*_*0*_ = 0.7, *NA* = 14.2 [37]).

We observed that genetic differentiation among mainland Australian populations is low, in contrast to island populations that were substantially differentiated among each other and from mainland populations. This population structure is most likely explained by relative isolation of islands compared to mainland populations. Exceptions from this general pattern are explained by human activities and their main pathways of trading and exploitation. Our results showed that the DHI population exhibits a relatively high genetic diversity (*N* = 39, haplotype diversity = 0.59, nucleotide diversity = 0.0018) and is genetically distinct except for some of the nearest mainland populations (PE, FG and MK, Table 2). Between 1850 and 1920 pearling was at its peak in the Shark Bay area, resulting in housing of workers on Dirk Hartog Island and the Peron Peninsula [23, 38]. Archeological remains indicate a large impact of Malaysian workers operating on pearling vessels and historical records state exchange between their homes in Malaysia and Shark Bay, Western Australia [38, 39]. The first report of a cat on a pearling lugger (to prevent seabirds roosting) was recorded on Dirk Hartog Island in 1920 [24, 40]. Later, cats were assumed to have been brought over during the time that the island was used as a pastoral sheep and goat station [41]. A recent study showed regular gene flow between the Western Australian mainland and DHI during the last decade, which has now ceased [42]. Since the introduction of cats to the island, 10 of the 13 native terrestrial mammals once present are now locally extinct, most probably due to the predation by cats [16, 43, 44].

The scenario selected in the model selection approach showed dispersal of cats from Europe to Australia and secondary introductions leading to gene flow between Cocos and Christmas Island, Asia and Australia (Fig. 2). Cocos Island was inhabited around 1820 by European merchants accompanied by Malaysian workers [30, 45]. One of the merchants built a settlement on Christmas Island supplying the growing industry on Cocos Island (i.e. with timber and provisions) while travelling regularly between Singapore and the two islands [31, 46, 47]. Extensive travel between Australia, Cocos and Christmas Island as well as Southeast Asia [31, 46, 47] is consistent with the results of the model selection approach for cat introductions over the past 200 years.

Cats from Dirk Hartog Island are found in several subgroups of the mitochondrial phylogenetic tree, together with West Australian localities (Fig. 3). Thus, these Dirk Hartog Island cats were likely of European or mixed European-Asian origin deriving from populations in Western Australian settlements which themselves originated possibly from shipwrecks around 1600 and definitely with substantial European visitation since 1850 [23–25, 44]. The main introduction of cats onto Dirk Hartog Island happened, at latest, during its main use as a pearling site around 1850 and 1920. Therefore, we can suggest multiple invasions of cats in Western Australia from Europe and Southeast Asia in the 19th century, providing a timeframe for the impact of feral cats on native species. Our greater understanding about the history of Australian feral cats may help to assess the relative impact of other non-native predators (namely the dingo, *Canis lupus dingo* and European fox) on native species prior and post European settlement [48, 49].

The patterns of human colonization are mirrored in cat genetic data from Tasmanian populations (TAS) and its neighbouring islands, Tasman Island (TASM) and Flinders Island (FL). Although these islands lie closely together, feral cats of TAS cluster (microsatellite and mitochondrial DNA analyses) into completely different groups from the cats of TASM and FL (Figs. 2 and 3). At the beginning of the 19th century cats were introduced to Tasmania during European settlement together with various workers (including Asians) of numerous industries [50–54]. The settlement and these industries would have resulted in regular visits to Tasmania, Tasman and Flinders Islands, by ships and traders on their way to the Australian mainland, European or Asian locations. Feral cats were present on Tasman Island following the construction of the lighthouse and eradicated in 2010 [55–57]. Cats on FL might have been present since the early 19th century with a small settlement established by sealers, later used to exile the remnants of the Tasmanian aboriginal human population. TASM and FL did not experience a major human influx from Europe or Asia (TASM now unpopulated, FL population approx. 776; Census, Australian Bureau of Statistics, 2011). Feral cats on the islands have therefore been more or less isolated from interbreeding with domestic fancy cat breeds being introduced as house pets. In contrast, TAS has been populated by up to 495,000 people (Census, Australian Bureau of Statistics, 2011) since the first settlement. In 1995 the Australian Bureau of Statistics estimated that 26.7 % of pet owners had cats as household pets and 17.5 % of the households in TAS reported problems with stray and feral cats (Australian Social Trends, 1995, Australian Bureau of Statistics). Previous studies have documented the extensive predatory impact of stray and feral cats on native fauna in suburban, rural and pastoral areas of Australia and indicated the possibility of intermixing between stray/domestic and feral cats [13, 58]. Clearly, Tasmania must be affected in both ways. Therefore, we should take into account that large numbers of fancy breed and domestic cats from the Australian mainland were brought onto the island intermixing with the original feral cats. This is also supported by the low genetic differentiation between Tasmania and Tips South West (TSW) (Table 2), since TSW represents a mixture of stray, feral, domestic and fancy breed cats. Although all three islands (TAS, TASM and FL) were among the first islands on which cats were known to be introduced [24], only Flinders Island and Tasman Island are genetically differentiated from all other Australian populations. We hypothesize that these populations consist of the descendants of the original invading lineages during the 19th century. In contrast to many other Australian populations, they remained largely isolated from subsequent mixing. Thus these island populations provide valuable information to trace back the global invasion routes of cats. Interestingly, cats from Flinders and Tasman Islands have close affinity, in terms of microsatellites, with the Cocos Islands. These microsatellite characteristics may thus be representative of the early colonizing cats according to best supported migration model (Fig. 2).

## Conclusion

Our results indicate a mainly European origin of feral cats in Australia with possible secondary introductions from Asia following the initial establishment of cats in Australia. Although this reflects the best-supported model by the model selection approach, models tested were limited to those from a series thought reasonably likely to represent regional history.

With regards to colonization history, it should be emphasised that cats on the islands surrounding Australia are of particular interest and may represent founding populations. Taken together with historical record, the genetic data suggest introduction of cats to Australia mainly following European settlements, providing an important timeframe for the impact of feral cats on native species in Australia. Further precision may be possible with more detailed (genomic scale) genetic data and a search for archaeological specimens, which themselves may be subject to genetic analysis.

## Methods

Cats were sampled across Australia, Southeast Asia and surrounding islands (Additional file 1: Table S3); these were feral except for Tips South West (TSW) and Malaysia. TSW individuals represent house or stray cats including descendants of fancy breeds; they were caught at rubbish tips. Malaysian samples were collected from a mixture of feral and stray cats with only hair samples taken. No formal ethical approvals of Malaysian authorities were required, since sampling was fully non-invasive (gentle tugging of fur). Trapping and collection of tissue samples from cats was conducted as described in [42]. This research had full ethical approvals for all techniques used by the Department of Parks and Wildlife (DPaw) Wildlife Animal Ethics Committee (AEC numbers: DEC AEC 2006-06, DEC AEC 2009-35 and DPaW AEC 2012–41). All samples collected were of ownerless cats. Blood or hair samples were taken as appropriate using NucleoSave Cards (Macherey-Nagel).

### DNA extraction, genotyping and sequencing

DNA was isolated using the NucleoSpin Tissue Kit (Macherey-Nagel) for tissue and blood samples and the ChargeSwitch Forensic DNA Purification Kit (Invitrogen) for hair samples.

The molecular work followed closely our previous protocols [42]. We genotyped most samples at the same 12 microsatellite loci, including a gender-identifying sequence tagged site from the Y-chromosome *SRY* gene [42, 59, 60]. We also obtained DNA sequences of the mitochondrial *ND5* and *ND6* gene regions for comparison with a previously published dataset by Driscoll et al. [19]. The mitochondrial segment was sequenced using a Biorad C1000 Thermocycler following the protocol of [42]. DNA sequences were determined using an ABI 3730 sequencer and analysed using Geneious 5.6.6 (Biomatters) software and Genemarker V1.95 (Softgenetics) software for microsatellites.

### Genetic variation and structure

A total of 269 feral cat mtDNA sequences representing the Australian mainland and island populations as well as Asian populations (hereafter referred to as the Australasian dataset) were analysed together with a subset of 42 sequences from European locations published by Driscoll et al. [19] (Additional file 1: GenBank: [EF587077.1-EF587153.1], Table S3B). European samples were selected to cover a broad geographic range and match quality criteria. Mitochondrial genetic diversity of the Australasian dataset and European populations was based on the number of haplotypes, haplotype diversity (*h*) and nucleotide diversity (*π*) using DNASP V5.1 [61]. Pairwise *F*_*ST*_ values were calculated using ARLEQUIN 3.5 [62].

A Bayesian phylogenetic tree was reconstructed using Beast v1.7.5 [63]. The analysis was run 5 × 10^7^ MCMC generations, sampling every 1000th generation. Log files were analysed using Tracer v1.5, to assess convergence and to confirm combined effective sample size (ESS) >200 for each parameter. A maximum credibility tree was subsequently produced using TreeAnnotator v1.6.1. FigTree v1.4.0 was used to graphically display the tree.

Microsatellite data were examined for null alleles using Microchecker [32]. GENEPOP 4.0 software [64] was used for the Australasian dataset to calculate basic population genetic parameters: mean number of alleles per locus (*NA*); expected (*H*_*E*_) and observed (*H*_*O*_) heterozygosity as well as significance values for deviations from Hardy-Weinberg equilibrium (HWE). Allele frequencies and *F*_*IS*_ coefficients as a measure of the level of inbreeding were calculated using FSTAT 2.9.3 [65]. Populations below sample size 10 were excluded from population genetic analysis (Table 1). To conduct analysis of molecular variance (AMOVA) in ARLEQUIN 3.5 [62] samples were grouped according to four main geographic regions: 1) Australian mainland, Dirk Hartog Island, Tasmania (OZ); 2) Cocos Island and Christmas Island (CIQ); 3) Europe (EU); 4) Asia (AS).

Fine-scale population structure was examined by determining the number of private alleles in each population. A principal coordinate analysis (PCoA) was conducted on a pairwise distance matrix for individuals GENALEX 6.5; [66]. Ancestry structure among the Australasian populations was studied with STRUCTURE 2.3.4 [67]. Individuals were assigned to clusters using an unbiased Bayesian approach under an admixture model. Burn-in and MCMC iteration settings were 50,000 and 100,000, respectively. Runs for each *K* were repeated 10 times. The best supported number of clusters based on the Δ*K* statistic was estimated using STRUCTURE Harvester v 0.6.93 [68]. The software CLUMPP [69] was used to align multiple replicates for *K* and the DISTRUCT application [70] was used to display the results graphically.

### Phylogeographic Model Selection (PMS)

We used MIGRATE-N 3.4 [71] to choose among competing dispersal hypotheses [72]. Two hundred and nine mitochondrial sequences of 24 sampling sites were pooled into four geographic groups (Europe, EU; Malaysia/Sulawesi, AS; Christmas/Cocos Island, CIQ; Australia, OZ). Considering EU, AS and CIQ as possible sources of colonization of OZ, we developed eleven phylogeographic hypotheses, based on historical possibilities, each of which had a corresponding maximum likelihood migration rate matrix (Additional file 1: Table S2, Additional 4: Figure S3). The starting parameters were adapted from Jesse et al. [73]. We ran a burn-in phase of 10,000 generations and ten short chains with 50,000 generations each, of which every 5,000th tree was recorded. Three long chains of 500,000 generations followed, from which 1,000 trees were sampled after burn-in of 50,000 generations. The transition/transversion ratio was set to 12.8 after estimation using DNASP V5.1. [61]. A final analysis with an unconstrained migration model using a likelihood-ratio-test was performed and Akaike Information Criterion (AIC) scores were obtained for each model.

### Availability of supporting data

The microsatellite data sets supporting the results of this article are available in the Dryad repository [Dryad doi:10.5061/dryad.6t066 (http://dx.doi.org/10.5061/dryad.6t066)]. All sequence data is available on GenBank, [GenBank accessions: KP279467 - KP279629, http://www.ncbi.nlm.nih.gov/genbank].

## Additional files

Additional file 1: Table S1. Results of the Bayesian assignment approach using STRUCTURE based on the cat microsatellite data. Shown are the mean posterior probabilities of *K* as well as the standard deviation, log likelihood of *K*, second order rate of change of log likelihood and Delta *K*. Preferred *K* value (highest Delta *K*) is shown in bold. Reps means number of repetitions for each *K*. **Table S2.** Results of the phylogeographic hypothesis model selection as applied to the mitochondrial *ND5* + *ND6* data for movements between Europe (EU), Australia (OZ), Christmas and Cocos (Keeling) Island (CIQ) and Malaysia/Sulawesi (AS) (detailed information of phylogeographic models, Additional file 4: Figure S3). AIC values measure the fit of the model to the data, taking different parameterisation into account. Smaller values indicate a better fit. The model with the best fit is shown in bold. **Table S3.** A. List of sample locations with abbreviations for sample location and region as well as number of specimens and corresponding geographical coordinates. B. List of European mitochondrial dataset published by Driscoll et al. (2007) [19] with accession numbers and abbreviation for sample region. (PDF 216 kb) Additional file 2: Figure S1. STRUCTURE bar plots showing *K* values (*K* = 2 and *K* = 3) below the optimal one, inferred from microsatellite data for mainland Australia, Australian islands and Southeast Asia. In each plot, each cluster is represented by a different colour, and each individual cat is represented by a vertical line divided into *K* coloured segments with heights proportional to genotype memberships in the clusters. Thin black lines separate individuals from different populations. Abbreviations for populations follow Table 1. (PDF 552 kb) Additional file 3: Figure S2. Phylogenetic tree of cats based on mtDNA haplotypes obtained in this paper together with those of Driscoll et al. 2007, reconstructed by Bayesian inference with 95 % highest posterior density (HPD) represented at nodes. (PDF 2542 kb) Additional file 4: Figure S3. Figures illustrating the phylogeographic model selection as applied to the mitochondrial ND5 + ND6 between Europe (EU), Australia (OZ), Christmas and Cocos (Keeling) Island (CIQ) and Malaysia/Sulawesi (AS). (PDF 1909 kb)
