# Supplementary material for: A voyage to Terra Australis: human-mediated dispersal of cats
Source: BMC Evol Biol. 2015 Dec 4;15:262. doi: 10.1186/s12862-015-0542-7 (PMC4669658; doi:10.1186/s12862-015-0542-7)
Supplement: Additional file 1: Table S1. — Results of the Bayesian assignment approach using STRUCTURE based on the cat microsatellite data. Shown are the mean posterior probabilities of K as well as the standard deviation, log likelihood of K, second order rate of change of log likelihood and Delta K. Preferred K value (highest Delta K) is shown in bold. Reps means number of repetitions for each K. Table S2. Results of the phylogeographic hypothesis model selection as applied to the mitochondrial ND5 + ND6 data for movements between Europe (EU), Australia (OZ), Christmas and Cocos (Keeling) Island (CIQ) and Malaysia/Sulawesi (AS) (detailed information of phylogeographic models, Additional file 4: Figure S3). AIC values measure the fit of the model to the data, taking different parameterisation into account. Smaller values indicate a better fit. The model with the best fit is shown in bold. Table S3. A. List of sample locations with abbreviations for sample location and region as well as number of specimens and corresponding geographical coordinates. B. List of European mitochondrial dataset published by Driscoll et al. (2007) [19] with accession numbers and abbreviation for sample region. (PDF 216 kb) [file 12862_2015_542_MOESM1_ESM.pdf]

Table S1. Results of the Bayesian assignment approach using STRUCTURE based on the cat microsatellite data. Shown are the mean posterior probabilities of *K* as well as the standard deviation, log likelihood of *K*, second order rate of change of log likelihood and Delta *K*. Preferred *K* value (highest Delta *K*) is shown in bold. Reps means number of repetitions for each *K*.

K	Reps	Mean LnP(*K*)	Stdev LnP(*K*)	Ln'(*K*)	|Ln''(*K*)|	Delta *K*
2	10	-90.991	1.669	-	-	-
3	10	-86.711	413.355	427.930	564.040	13.645
**4**	**10**	**-88.072**	**4.679**	**-136.110**	**12.537**	**26.795**
5	10	-214.813	196.401	-12.674	7.667	0.390
6	10	-264.874	527.213	-5.006	19.947	0.378
7	10	-115.465	89.350	14.940	-	-

Table S2. Results of the phylogeographic hypothesis model selection as applied to the mitochondrial *ND5* + *ND6* data for movements between Europe (EU), Australia (OZ), Christmas and Cocos (Keeling) Island (CIQ) and Malaysia/Sulawesi (AS) (detailed information of phylogeographic models, Additional file, Figure S3). AIC values measure the fit of the model to the data, taking different parameterisation into account. Smaller values indicate a better fit. The model with the best fit is shown in bold.

Scenario	No. of parameters	LnL	AIC	delta AIC
Model 1	7	-953.24	1920.49	195.78
Model 2	9	-953.24	1924.49	199.78
Model 3	11	-927.87	1877.75	153.05
Model 4	10	-876.68	1773.37	48.66
Model 5	13	-851.35	1728.70	4
Model 6	8	-876.68	1769.37	44.66
Model 7	10	-876.68	1773.37	48.66
Model 8	9	-876.68	1771.37	46.66
Model 9	11	-876.68	1775.37	3500.07
**Model 10**	**11**	**-851.35**	**1724.70**	**0**
Model 11	8	-927.87	1871.75	147.05

Table S3. A. List of sample locations with abbreviations for sample location and region as well as number of specimens and corresponding geographical coordinates. B. List of European mitochondrial dataset published by Driscoll et al. (2007) with accession numbers and abbreviation for sample region.

A

Location	Sample location abbreviation	Abbreviation for region	Number of specimens	Latitude/ longitude
Christmas Island	CIF	CIQ	79	10.48396 S, 105.635794 E
Cocos (Keeling) Island	Q	CIQ	42	12.176745 S, 96.819696 E
Malaysia	M	AS	17	5.834056 N, 116.176552 E
Sulawesi	SU	AS	3	0.946428 N, 122.338332 E
Tasmania	TAS	OZ	10	42.330398 S, 146.066151 E
Flinders Island	FL	OZ	3	39.841386 S, 147.927579 E
Tasman Island	TASM	OZ	5	43.239045 S, 148.002957 E
Tips South West	TSW	OZ	25	33.363746 S, 116.183907 E
Cape Arid	CA	OZ	23	33.652099 S, 123.371850 E
Portman	P	OZ	3	30.02964 S, 119.48.2748 E
Victoria	VIC	OZ	5	36.630151 S, 147.239027 E
Peron Peninsula	PE	OZ	13	25.759409 S, 113.454491 E
Fitzgerald National Park	FG	OZ	10	33.889118 S, 119.883647 E
French Island	FI	OZ	3	38.295343 S, 145.318245 E
Kimberley	KIM	OZ	5	17.737799 S, 126.400367 E
Mount Keith	MK	OZ	14	27.260694 S, 120.510971 E
Dirk Hartog Island	DHI	OZ	40	25.791562 S, 113.038384 E

B

Sample ID	Location	Abbreviation for region	Accession number
7077Fra	France	EU	EF587077.1
7081Fra	France	EU	EF587081.1
7084Fra	France	EU	EF587084.1
7158Fra	France	EU	EF587158.1
7131Fra	France	EU	EF587131.1
7132Fra	France	EU	EF587132.1
7155Fra	France	EU	EF587155.1
7086Fra	France	EU	EF587086.1
7133Fra	France	EU	EF587133.1
7166Fra	France	EU	EF587166.1
7138Ger	Germany	EU	EF587138.1
7152Ser	Serbia	EU	EF587152.1
7160Ser	Serbia	EU	EF587160.1
7162Port	Portugal	EU	EF587162.1
7164Port	Portugal	EU	EF587164.1
7130Hun	Hungary	EU	EF587130.1
7149Hun	Hungary	EU	EF587149.1
7167Hun	Hungary	EU	EF587167.1
7140Spai	Spain	EU	EF587140.1
7159Spai	Spain	EU	EF587159.1
7163Spai	Spain	EU	EF587163.1
7168Spai	Spain	EU	EF587168.1
7169Spai	Spain	EU	EF587169.1
7170Spai	Spain	EU	EF587170.1
7172Spai	Spain	EU	EF587172.1
7174Spai	Spain	EU	EF587174.1
7100Bah	Bahrain	EU	EF587100.1
7101Bah	Bahrain	EU	EF587101.1
7102Bah	Bahrain	EU	EF587102.1
7097Eng	England	EU	EF587097.1
7099Eng	England	EU	EF587099.1
7103Eng	England	EU	EF587103.1
7105Eng	England	EU	EF587105.1
7117Eng	England	EU	EF587117.1
7122Eng	England	EU	EF587122.1
7126Eng	England	EU	EF587126.1
7039Scot	England	EU	EF587039.1
7040Scot	England	EU	EF587040.1
7153Scot	England	EU	EF587153.1
