# Supplementary material for: A voyage to Terra Australis: human-mediated dispersal of cats
Source: BMC Evol Biol. 2015 Dec 4;15:262. doi: 10.1186/s12862-015-0542-7 (PMC4669658; doi:10.1186/s12862-015-0542-7)
Supplement: Additional file 2: Figure S1. — STRUCTURE bar plots showing K values (K = 2 and K = 3) below the optimal one, inferred from microsatellite data for mainland Australia, Australian islands and Southeast Asia. In each plot, each cluster is represented by a different colour, and each individual cat is represented by a vertical line divided into K coloured segments with heights proportional to genotype memberships in the clusters. Thin black lines separate individuals from different populations. Abbreviations for populations follow Table 1. (PDF 552 kb) [file 12862_2015_542_MOESM2_ESM.pdf]

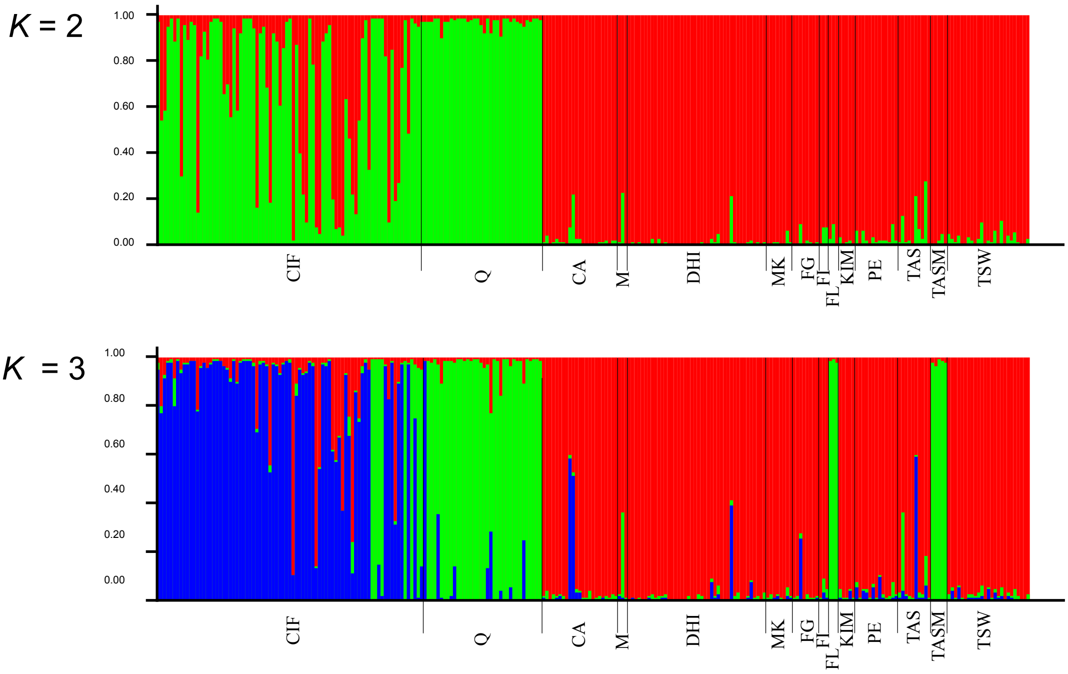

Figure S1. STRUCTURE bar plots showing *K* values (*K* = 2 and *K* = 3) below the optimal one, inferred from microsatellite data for mainland Australia, Australian islands and Southeast Asia. In each plot, each cluster is represented by a different colour, and each individual cat is represented by a vertical line divided into *K* coloured segments with heights proportional to genotype memberships in the clusters. Thin black lines separate individuals from different populations. Abbreviations for populations follow Table 1.
