# Supplementary figures and images for: A voyage to Terra Australis: human-mediated dispersal of cats
Source: BMC Evol Biol. 2015 Dec 4;15:262. doi: 10.1186/s12862-015-0542-7 (PMC4669658; doi:10.1186/s12862-015-0542-7)

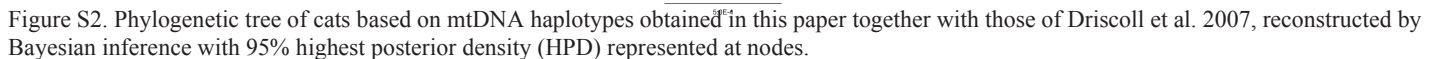

Supplement: Additional file 3: Figure S2. — Phylogenetic tree of cats based on mtDNA haplotypes obtained in this paper together with those of Driscoll et al. 2007, reconstructed by Bayesian inference with 95 % highest posterior density (HPD) represented at nodes. (PDF 2542 kb) [file 12862_2015_542_MOESM3_ESM.pdf]
