# Supplementary material for: A voyage to Terra Australis: human-mediated dispersal of cats
Source: BMC Evol Biol. 2015 Dec 4;15:262. doi: 10.1186/s12862-015-0542-7 (PMC4669658; doi:10.1186/s12862-015-0542-7)
Supplement: Additional file 4: Figure S3. — Figures illustrating the phylogeographic model selection as applied to the mitochondrial ND5 + ND6 between Europe (EU), Australia (OZ), Christmas and Cocos (Keeling) Island (CIQ) and Malaysia/Sulawesi (AS). (PDF 1909 kb) [file 12862_2015_542_MOESM4_ESM.pdf]

Model 1

	ciq	oz	eu	as
ciq	x	0	0	0
oz	0	x	0	0
eu	x	x	x	x
as	0	0	0	x

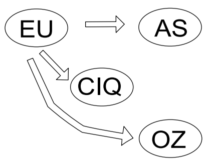

Model 2

	ciq	oz	eu	as
ciq	x	x	0	0
oz	0	x	0	0
eu	x	x	x	x
as	0	x	0	x

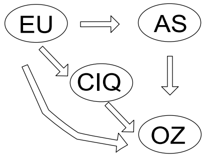

Model 3

	ciq	oz	eu	as
ciq	x	x	0	0
oz	x	x	0	x
eu	x	x	x	x
as	0	x	0	x

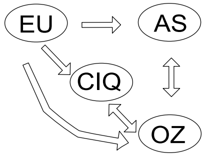

Model 4

	ciq	oz	eu	as
ciq	x	x	0	0
oz	0	x	0	0
eu	x	x	x	x
as	x	x	0	x

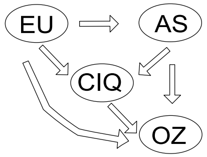

Model 5

	ciq	oz	eu	as
ciq	x	x	0	x
oz	x	x	0	x
eu	x	x	x	x
as	x	x	0	x

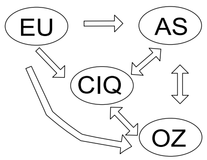

Model 6

	ciq	oz	eu	as
ciq	x	x	0	0
oz	0	x	0	0
eu	0	x	x	x
as	x	0	0	x

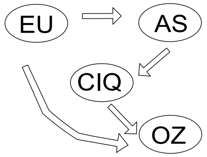

Model 7

	ciq	oz	eu	as
ciq	x	x	0	x
oz	x	x	0	0
eu	0	x	x	x
as	x	0	0	x

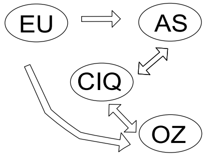

Model 8

	ciq	oz	eu	as
ciq	x	x	0	0
oz	0	x	0	0
eu	x	x	x	x
as	x	0	0	x

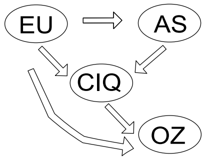

Model 9

	ciq	oz	eu	as
ciq	x	x	0	x
oz	x	x	0	0
eu	x	x	x	x
as	x	0	0	x

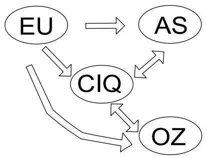

Model 10

	ciq	oz	eu	as
ciq	x	x	0	x
oz	x	x	0	x
eu	0	x	x	0
as	x	x	0	x

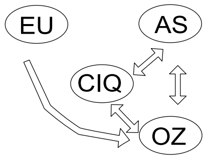

Model 11

	ciq	oz	eu	as
ciq	x	0	0	x
oz	x	x	0	x
eu	0	x	x	0
as	0	0	0	x

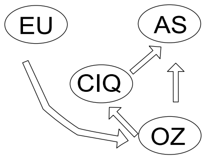

Figure S3. Figures illustrating the phylogeographic model selection as applied to the mitochondrial ND5 + ND6 between Europe (EU), Australia (OZ), Christmas and Cocos (Keeling) Island (CIQ) and Malaysia/Sulawesi (AS).
